# Molecular Characterization of Cancer Preventive and Therapeutic Potential of Three Antistress Compounds, Triethylene Glycol, Withanone, and Withaferin A

**DOI:** 10.3390/ijms26020493

**Published:** 2025-01-09

**Authors:** Huayue Zhang, Hyonchol Kim, Tian Yuan, Zhenya Zhang, Sunil C. Kaul, Renu Wadhwa

**Affiliations:** 1 Graduate School of Science and Technology, University of Tsukuba, Ibaraki 305-8575, Japan; s2130297@u.tsukuba.ac.jp (H.Z.); yuan.tian.ga@u.tsukuba.ac.jp (T.Y.); zhang.zhenya.fu@u.tsukuba.ac.jp (Z.Z.); 2 AIST-INDIA DAILAB, National Institute of Advanced Industrial Science & Technology (AIST), Central 4-1, Tsukuba 305-8565, Japan; kim-hc@aist.go.jp (H.K.); s-kaul@aist.go.jp (S.C.K.)

**Keywords:** triethylene glycol, Withanone, Withaferin-A, cancer stem cells, inhibition, differentiation therapy

## Abstract

The molecular link between stress and carcinogenesis and the positive outcomes of stress intervention in cancer therapy have recently been well documented. Cancer stem cells (CSCs) facilitate cancer malignancy, drug resistance, and relapse and, hence, have emerged as a new therapeutic target. Here, we aimed to investigate the effect of three previously described antistress compounds (triethylene glycol, TEG; Withanone, Wi-N, and Withaferin A, Wi-A) on the stemness and differentiation characteristics of cancer cells. Breast carcinoma, glioblastoma, and neuroblastoma cells were treated with a non-toxic concentration of TEG (0.1%), Wi-N (5 µM), and Wi-A (0.1 µM) in 2D and 3D cultures. The results demonstrated that TEG, Wi-N, and Wi-A suppressed the stemness properties, which was linked with their inhibition of epithelial–mesenchymal transition (EMT) signaling. In particular, Wi-N and TEG caused a stronger reduction in the self-renewal capability of CSCs than Wi-A, as evidenced by a tumor spheroid formation assay and analyses of stemness-related genes (*ALDH1*, *CD44*, *NANOG*, *CD133*, *SOX2*). Furthermore, TEG and Wi-N caused the differentiation of cancer cells. Each of these was supported by (i) the upregulation of *KRT18*, *KRT19*, *E-cadherin*, and downregulation of *vimentin* in breast carcinoma; (ii) increased levels of GFAP, MAP2, and PSD-95 in astrocytoma; and (iii) increased NeuN, GAP-43, and NF200 levels in neuroblastoma. Furthermore, a reduction in cancer progression-related proteins (PI3K, N-myc) was recorded in treated cells. Our results suggest that TEG and Wi-N may be recruited to target cancer cell stemness and differentiation therapy.

## 1. Introduction

Cancer, one of the leading mortality factors of age-related diseases, has attracted significant attention with increasing human lifespan and increasing aging populations over the last century. The recent literature has revealed several hallmarks of cancer, such as sustained proliferation, genomic instability, loss of tumor suppressor and gain of oncogenic functions, epigenetic alterations, activated metastasis, and inflammation, suggesting cancer as a chronic disease involving multistep processes [1,2]. Notably, these “cancer-enabling characteristics” largely overlap with the hallmarks of environmental stress insults [3], supporting the general notion that stress represents an essential factor that affects cancer initiation and progression [4]. Studies have indicated that specific stress factors contribute to cancer initiation via adrenergic receptor-mediated genome instability and tumor-promoting inflammation. Bernhard et al. reported that the β2-adrenergic receptor (ADRB2) and nerve growth factor (NGF)-brain-derived neurotrophic factor (BDNF)/Trk pathways are central to pancreatic cancer biology [5]. β-adrenergic receptor-mediated generation of reactive oxygen species (ROS) and β-arrestin–MDM2-dependent p53 degradation led to the accumulation of DNA damage in non-cancer cell lines [6]. Huan et al. reported that the activation of α1-adrenergic receptors enhanced the activation of liver-resident macrophages, sustaining the inflammatory microenvironment during diethylnitrosamine-induced hepatocarcinogenesis in rats [7]. To date, the effects of stress on facilitating cancer progression through modulating survival, migration, invasion, angiogenesis, and epithelial–mesenchymal transition (EMT) signaling have been well documented [8,9,10,11]. In addition, stress has been reported to impair adjuvant and neoadjuvant cancer treatments [12,13]. Accordingly, the positive effects of stress interventions on cancer therapy can be predicted.

On the other hand, conventional cancer therapies (radiotherapy and chemotherapy that aim at killing the actively proliferating tumor cells) often fail due to three lethal characteristics of cancer, including metastasis, acquired drug resistance, and tumor relapse. All these attributes have been closely linked to a subpopulation of undifferentiated cancer cells with strong self-renewal properties, also called cancer stem cells (CSCs) [14]. CSCs possess abnormally activated Wnt/β-catenin, Notch, PI3K/Akt/mTOR, TGF-β/SMAD, and NF-κB signaling, leading to imbalanced proliferation and malignant transformation [15,16,17,18,19]. Highly expressed stem cell marker proteins (e.g., Oct4, CD44, Nanog) and multidrug resistance proteins (MRPs) in CSCs reduce their sensitivity to cell death signaling, often leading to resistance to conventional toxic therapies [14,20]. Moreover, the activation of EMT signaling in these cells leads to metastasis and lineage switching, which help cancer cells transition into a type resistant to the original therapy, often resulting in treatment failure [21,22].

Meanwhile, CSCs possess a high potential to differentiate and thus draw attention to differentiation therapy that can abrogate CSC self-renewal and activate antitumor immune responses [23]. Clinical studies have reported a high success rate (complete remission in ~85% of cases) in all-trans retinoic acid-based differentiation therapy for acute promyelocytic leukemia [24]. Similarly, bromodomain-containing protein 4 (BRD4) and histone deacetylase (HDAC) inhibitors were reported to trigger differentiation and tumor regression in xenograft models or patients [25,26]. Differentiation therapy in leukemia, melanoma, glioblastoma, and breast carcinoma with cAMP, peroxisomal proliferator receptor-γ (PPARγ) agonists, and isocitrate dehydrogenase (IDH) inhibitors in in vitro models has also been documented [27,28,29,30]. Furthermore, inducing differentiation instead of death in cancer cells can avoid adverse effects such as physical and psychological stress, especially in elderly patients [2]. Using natural compounds can further help improve the therapeutic response and sustain quality of life during and post-treatment [31]. For example, curcumin was reported to induce the differentiation of myeloid-derived suppressor cells and inhibit related tumor growth. Dietary curcumin reduced colonic tumor burden associated with increased richness of gut Lactobacillales in *Il*10^−/−^ mice [32,33]. Emerging research on herbal extracts such as Ashwagandha (*Withania somnifera*) extracts and wheatgrass juice has revealed their adjuvant efficacy towards cancer treatment by promoting differentiation and reshaping the tumor microenvironment [34,35]. Overall, findings from different approaches strongly suggest the possibility of recruiting differentiation therapy alone or in combination with traditional therapies to eradicate CSCs and avoid treatment failure [22].

Recently, we identified three antistress compounds: triethylene glycol (TEG), Withanone (Wi-N), and Withaferin A (Wi-A) in cell culture-based assays. Molecular assays revealed that non-toxic doses of these compounds protected cultured cells against oxidative, metal, and hypoxia stress-induced apoptosis, reactive oxygen species (ROS) accumulation, mitochondrial depolarization, DNA damage, and protein aggregation. Meanwhile, TEG, Wi-N, and their mixture showed an antiaging effect in the replicative senescence model, as reflected by the decrease in DNA damage and promotion of fibroblast proliferation [36]. Both Wi-A and Wi-N are steroidal lactones often isolated from Ashwagandha extracts. Wi-A is a C5, C6 epoxy compound carrying hydroxyl groups on C4 and C27, while Wi-N is a C6, C7 epoxy compound having hydroxyl groups on C5 and C17 [37]. TEG is a dihydroxy alcohol shown to possess antimicrobial and antiviral activities. It has also been detected in the water extract of Ashwagandha leaves [38]. Previous studies have also demonstrated that TEG, Wi-N, and Wi-A cause selective toxicity to cancer cells by activating p53 and pRB tumor suppressor signaling [38,39,40]. However, most of these studies focused on the toxic doses of TEG, Wi-N, and Wi-A; their effect on CSC properties at low non-toxic doses remains unknown.

Based on the above premise, we investigated the effect of TEG, Wi-N, and Wi-A on cancer stemness and differentiation therapy using three in vitro models (breast carcinoma, glioblastoma, and neuroblastoma). Cells treated with non-toxic doses of TEG and Wi-N showed remarkable inhibition of metastasis activity, EMT signaling, CSC self-renewal, and differentiation induction. The findings suggest that the antistress compounds TEG and Wi-N may be helpful in cancer treatment and prevention.

## 2. Results

### 2.1. TEG, Wi-N, and Wi-A Inhibited Metastasis Characteristics of Cancer Cells

**Figure 1:**
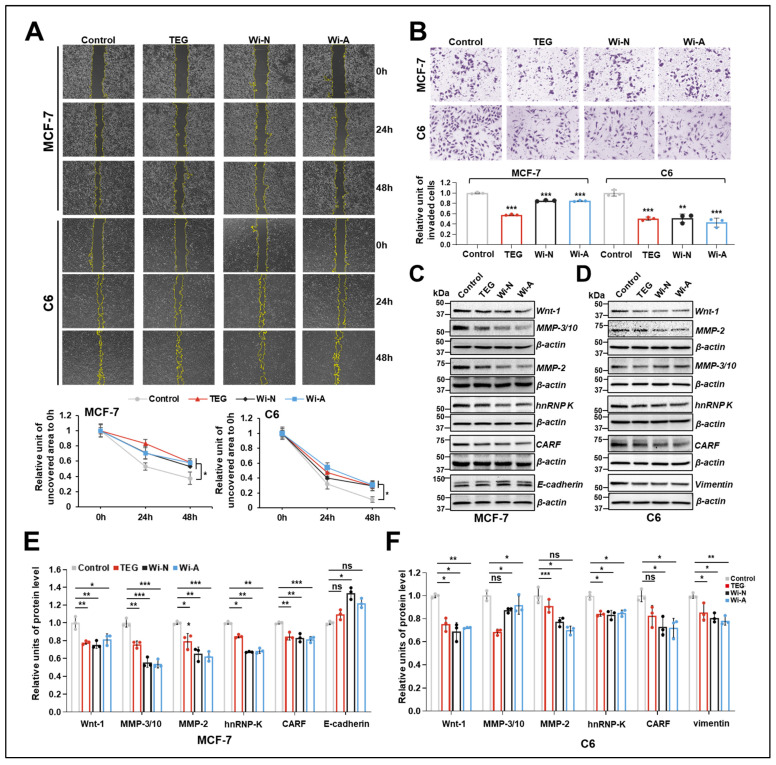
Inhibitory effect of TEG (0.1%), Wi-N (5 µM), and Wi-A (0.1 µM) on metastasis ability of cancer cells. (**A**) The wound healing assay showed decreased migration ability in TEG-, Wi-N-, and Wi-A-treated MCF-7 and C6 cells at 48 h. Images were captured at 4X magnification. Quantification from three independent experiments is shown below the images. (**B**) The Transwell assay showed decreased invasion activity in TEG-, Wi-N-, and Wi-A-treated MCF-7 and C6 cells at 48 h. Images were captured at 10× magnification. Quantification from three independent experiments is shown below the images. (**C** and **D**) Western blotting analysis showing inhibition of EMT signaling by TEG, Wi-N, and Wi-A treatment in MCF-7 (**C**) and C6 (**D**) cells at 48 h. (**E**,**F**) Quantification of Western blotting from three independent experiments. For (**A**), data were normalized against each group’s wound area of 0 h. For (**B**,**E**,**F**), data were normalized against the control group. All the data were plotted as fold difference (mean ± SD, n = 3). ^ns^ *p* ≥ 0.05, * *p* < 0.05, ** *p* < 0.01, *** *p*< 0.001 denote statistical significant differences from the control group (one-way ANOVA with Dunnett’s multiple comparisons). Control: 0.05% DMSO; ns: not significant; TEG: triethylene glycol; Wi-N: Withanone; Wi-A: Withaferin A.

In our previous study on screening for antistress compounds using C6 cells, we reported that TEG, Wi-A, and Wi-N have the capacity to protect cells against oxidative, metal, and hypoxia stresses [36]. To investigate the anti-metastasis potential of these compounds, we here used breast carcinoma MCF7, which also exhibits high cancer stemness characteristics. Non-toxic doses of the compounds were first selected by dose- and time-dependent cell viability assays. Treatment with higher concentrations used in this study, such as TEG (0.2%), Wi-N (10 µM), and Wi-A (0.2 µM) for 72 h, caused ~20% reduction in viability. Low concentrations such as TEG (0.1%), Wi-N (5 µM), and Wi-A (0.1 µM) caused less than 10% cytotoxicity for both MCF-7 and C6 cells in a 24–72 h treatment regime (Figure S1). Based on these observations, a wound-healing assay was performed for cultures treated with these low, non-toxic concentrations of the compounds for 48 h. As shown in Figure 1A, we observed a significant inhibition of cell migration in treated MCF-7 and C6 cells compared to the controls. The wound-healing assay on the control and treated highly malignant breast cancer cell line, MDA-MB-231, revealed a small but significant delay in migration in TEG- and Wi-N-treated cells (Figure S2A). Wi-A, on the other hand, did not show a significant effect. In light of these data, we performed the Transwell invasion assay in MCF-7 and C6 cells treated with the three compounds for 48 h, out of which TEG showed more potent inhibitory activity in both cell lines (Figure 1B).

To investigate the molecular mechanisms of such anti-migration and anti-invasion activities, we examined the molecular markers involved in EMT by Western blotting. As shown in Figure 1C–F, treated MCF-7 and C6 cells displayed a reduction in Wnt-1, hnRNP-K (heterogeneous nuclear ribonucleoprotein K), and CARF (Collaborator of ARF) proteins, coupled with a slight increase in E-cadherin level in Wi-N-treated MCF-7 cells and a remarkable decrease in Vimentin in treated C6 cells. In addition, matrix metalloproteinases (MMP-2 and MMP-3/10) were also reduced in both cell types. Consistent with the results of the wound-healing assay showing weak anti-migration activity of the three compounds in MDA-MB-231, molecular marker analyses showed only a small decrease in Vimentin in cells treated with the three compounds. On the other hand, no difference in hnRNP-K and MMP-2 levels was observed in control and treated cells (Figure S2B). These data suggested that the three test compounds (TEG, Wi-N, and Wi-A) exhibit good anti-metastasis potential through inhibition of EMT signaling in MCF-7 and C6 cells.

### 2.2. TEG, Wi-N, and Wi-A Caused a Reduction in Cancer Cell Stemness

**Figure 2:**
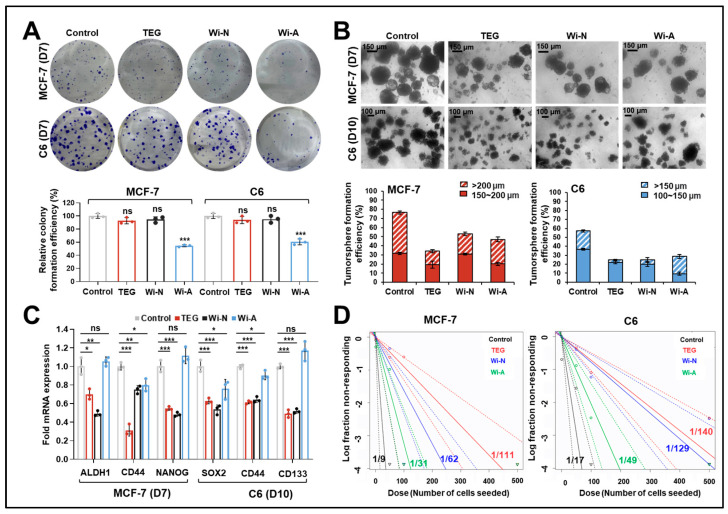
Attenuation of cancer stemness by TEG (0.1%), Wi-N (5 µM), and Wi-A (0.1 µM) treatment in MCF-7 and C6 cells. (**A**) The colony formation assay showed decreased colony formation efficiency in Wi-A but not in TEG- and Wi-N-treated cells on day 7. Quantification from three independent experiments is shown below the images. (**B**) The tumorsphere formation assay showed inhibited tumor spheroid formation efficiency in cells by TEG, Wi-N, and Wi-A treatment. Quantification from three independent experiments is shown below the images. The scale bars represent 150 µm in images of MCF-7 cells and 100 µm in images of C6 cells, respectively. (**C**) RT-qPCR analysis showing downregulation of cancer stemness-related markers in TEG-, Wi-N-, and Wi-A-treated spheroids. (**D**) Extreme limiting dilution assay showing a reduction in spheroid formation frequency of cells by TEG, Wi-N, and Wi-A treatment. Cells from the spheroids formed in tumorsphere formation experiments were seeded at a density of 500 cells/well to 1 cell/well and cultured for 7 days in a medium without indicated compounds. The numbers shown in the graph represent spheroid formation frequency. The dashed lines indicate the confidence interval. For (**A**), data were normalized against the control group and plotted as percentage difference (mean ± SD, n = 3). For (**C**), data were normalized against the control group and plotted as fold difference (mean ± SD, n = 3). ^ns^ *p* ≥ 0.05, * *p* < 0.05, ** *p* < 0.01, *** *p* < 0.001 denote statistical significant differences from the control group (one-way ANOVA with Dunnett’s multiple comparisons). Control: 0.05% DMSO; ns: not significant; TEG: triethylene glycol; Wi-N: Withanone; Wi-A: Withaferin A.

Since EMT is tightly linked with the maintenance of CSC properties, we next determined the effect of these compounds on cancer stemness. The colony formation assay was first performed to detect the long-term toxicity of low doses of the three compounds. Whereas Wi-A (0.1 µM) caused a reduction in colony number as well as size in both cell types, TEG (0.1%) and Wi-N (5 µM) treatments did not show any significant effect (Figure 2A). We then conducted the tumorsphere formation assay to assess the tumorigenic potential. The average number of positive spheres in control, TEG-, Wi-N-, Wi-A-treated cells was 51, 26, 40, and 34 for MCF-7 and 54, 31, 36, and 42 for C6 cultures, respectively. Further quantitative analysis revealed that TEG, Wi-N, and Wi-A decreased the tumorsphere formation efficiency to 34.1%, 53.1%, and 47%, respectively, compared to 76.7% in control MCF-7 cells. Similarly, C6 tumorspheres decreased from 57.2% (control) to 24.7%, 25%, and 28.7% upon TEG, Wi-N, and Wi-A treatments, respectively (Figure 2B).

Next, the spheroids formed in the control and treated groups of two cell lines were broken down and subjected to RT-qPCR to analyze stemness-related markers. As shown in Figure 2C, a remarkable reduction in *ALDH1*, *CD44*, and *NANOG* in MCF-7 cells and *SOX2*, *CD44*, and *CD133* in C6 cells were detected upon TEG and Wi-N treatments; Wi-A did not affect *ALDH1* and *NANOG* in MCF-7, and *CD133* in C6 cells. To further measure the ability of treated cells to reassemble into tumor spheroids, the spheroids were re-suspended into single-cell suspension in a medium without the compounds and then subjected to ELDA (Figure 2D). Consistently, the cells originating from TEG-, Wi-N-, and Wi-A-treated spheroids showed a lower frequency of spheroid formation (1/111, 1/62, and 1/31, respectively, in MCF-7; 1/140, 1/129, and 1/49, respectively, in C6) in comparison to control cells (1/9 in MCF-7 and 1/17 in C6 cells), suggesting inhibited self-renewal and tumor regenerative capacities in the treated cells. TEG, Wi-N, and Wi-A exhibited a decrease in cancer stemness, with TEG and Wi-N demonstrating higher effectiveness.

### 2.3. TEG, Wi-N, and Wi-A Induced Epithelial-like Differentiation in Breast Cancer Cells

**Figure 3:**
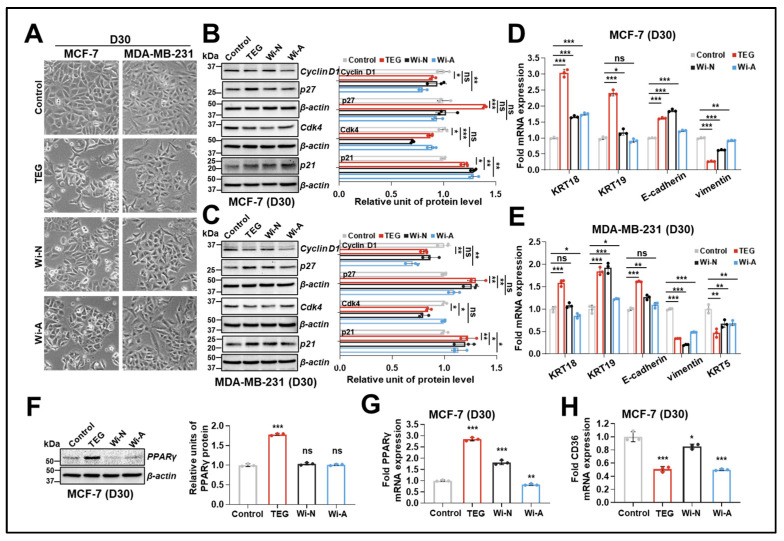
Induction of an epithelial-like differentiation by TEG (0.1%), Wi-N (5 µM) and Wi-A (0.1 µM) in breast carcinoma cells MCF-7 and MDA-MB-231. (**A**) Morphological observation of control and treated cells at day 30. Images were captured at 10X magnification. (**B**,**C**) Western blotting analysis showing different effects of TEG, Wi-N, and Wi-A on G0/G1 phase-related protein levels in MCF-7 (**B**) and MDA-MB-231 (**C**) cells. Quantitation from three independent experiments is shown on the right side. (**D**,**E**) RT-qPCR analysis shows upregulated epithelial and downregulated mesenchymal markers in treated MCF-7 (**D**) and MDA-MB-231 (**E**) cells. (**F**) Western blotting analysis of PPARγ showing induction of PPARγ by TEG in MCF-7 cells. Quantification from three independent experiments is shown on the right side. The PPARγ band was detected on the same blot of Cdk4 shown in (**B**). (**G**) RT-qPCR analysis of PPARγ showing upregulation by TEG and Wi-N and downregulation by Wi-A in MCF-7 cells. (**H**) RT-qPCR analysis showing TEG, Wi-N, and Wi-A downregulated *CD36* expression in MCF-7 cells. All data were normalized against the control group and plotted as fold difference (mean ± SD, n = 3). ^ns^ *p* ≥ 0.05, * *p* < 0.05, ** *p* < 0.01, *** *p* < 0.001 denote statistical significant differences from the control group (one-way ANOVA with Dunnett’s multiple comparisons). Control: 0.05% DMSO; ns: not significant; TEG: triethylene glycol; Wi-N: Withanone; Wi-A: Withaferin A.

To further validate the ability of these compounds to attenuate CSC properties, we subjected the cells to long-term (30-day) treatment. Microscopy observation of treated MCF-7 cells (Figure 3A) revealed a reduced cell number and a more flattened morphology with irregular boundaries. Similar results were obtained in MDA-MB-231. Biochemical characterization of control and treated cells with Western blotting for cell cycle-related proteins revealed a small but significant decrease in Cyclin D1 and Cdk4 and an increase in p27 and p21 levels in TEG-treated MCF-7 and MDA-MB-231 cells. Wi-N had a similar effect on Cdk4 and p21 levels, whereas Wi-A mainly influenced Cyclin D1 and p21 (Figure 3B,C). These data suggested that the compounds caused G1 phase arrest in MCF-7 and MDA-MB-231 cells. The control and treated cells were observed regularly for morphological changes. Although no signs of apoptosis were recorded, we examined the levels of apoptosis-related proteins in control and treated cells. As shown in Figure S3, we detected increased p53 protein in TEG- and Wi-N-treated MCF-7 (wild type p53) but not MDA-MB-231 (mutant p53) cells. The two cell lines showed an insignificant decrease in Bcl-2/Bax ratio and c-Myc levels in TEG- or Wi-N-treated cells. Of note, both Wi-A-treated MCF-7 and MDA-MB-231 cells showed a mild but significant reduction in Bcl-2/Bax ratio and c-Myc. However, none of the three compounds caused substantial change on the procaspase 3 level. The cleaved caspase was also not detected. These data endorsed a lack of apoptosis and the predominant occurrence of growth arrest in treated MCF-7 and MDA-MB-231 cells.

Next, we evaluated the transcriptional regulation of several epithelial and mesenchymal markers in control and treated cells. As shown in Figure 3D,E, RT-qPCR data revealed increased expression of epithelial/luminal markers *KRT18*, *KRT19*, and *E-cadherin*, as well as a reduction in expression of mesenchymal/basal markers *KRT5* (undetectable in MCF-7 cells) and *vimentin*, especially in TEG- and Wi-N-treated cultures, suggesting these cells gained more epithelial-like features. Interestingly, we observed a significant increase in PPARγ at protein (Figure 3F) and mRNA (Figure 3G) levels in TEG-treated MCF-7 cells. Besides, we also detected a decrease in *CD36* mRNA expression in treated MCF-7 cells (Figure 3H), which further supported the inhibition of proliferation. These data indicate that prolonged TEG and Wi-N treatment trigger epithelial-like differentiation, not apoptosis, in breast cancer cells.

### 2.4. TEG and Wi-N Caused Differentiation of Glioblastoma and Neuroblastoma

**Figure 4:**
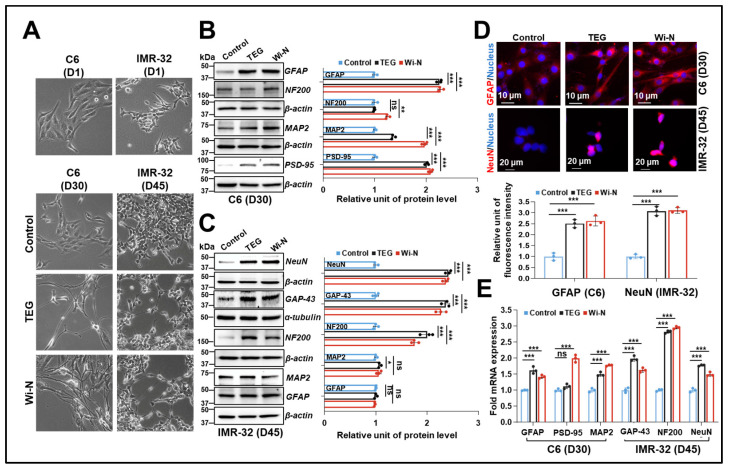
Induction of differentiation in glioblastoma cell C6 and neuroblastoma cell IMR-32 by TEG (0.1%) and Wi-N (5 µM). (**A**) Phase contrast images showing astrocytic and neuron-like morphology in TEG- and Wi-N-treated C6 (30 days, left side) and IMR-32 (45 days, right side) cells, respectively. Images were captured at 10X magnification. (**B**,**C**) Western blotting analysis showing increased levels of glial differentiation proteins GFAP, MAP2, and PSD-95 in treated C6 cells (**B**) and increased levels of neurodifferentiation proteins NeuN, GAP-43, NF200 in treated IMR-32 cells (**C**). Quantification from three independent experiments is shown on the right side. (**D**) Immunostaining of GFAP (in C6) and NeuN (in IMR-32) showed increased fluorescence intensity in treated cells. The scale bars represent 10 µm in images of C6 cells and 20 µm in images of IMR-32 cells, respectively. Quantification from three independent experiments is shown below the images. (**E**) RT-qPCR analysis shows upregulated *GFAP* and *MAP2* expression in treated C6 cells and upregulated *GAP-43*, *NF200*, and *NeuN* in treated IMR-32 cells. All data were normalized against the control group and plotted as fold difference (mean ± SD, n = 3). ^ns^ *p* ≥ 0.05, * *p* < 0.05, ** *p* < 0.01, *** *p* < 0.001 denote statistical significant differences from the control group (one-way ANOVA with Dunnett’s multiple comparisons). Control: 0.05% DMSO; ns: not significant; TEG: triethylene glycol; Wi-N: Withanone; Wi-A: Withaferin A.

Given the above findings, we further assessed the potential of TEG and Wi-N to induce differentiation in glioblastoma (C6) and neuroblastoma (IMR-32), two well-established cell models with high potential for astrocytes and neurons, respectively. Western blotting analysis post-96h-treatment showed increased levels of p21 and the differentiation marker GFAP in TEG-treated C6 cells. Cells treated with Wi-N showed an increase in p21 only (Figure S4A). Meanwhile, IMR-32 cells treated with TEG and Wi-N for 96 h showed no change in cell cycle and differentiation proteins compared to the control group (Figure S4B). However, both cell types showed differentiated morphology after long-term treatment. Distinct star-shaped C6 cells (30 days of culture) and mature neuron-like IMR-32 cells showing axonal extensions and reconnections (45 days of culture) were observed (Figure 4A). We also confirmed the differentiation state of the cells by Western blotting (Figure 4B,C). TEG- and Wi-N-treated C6 and IMR-32 cells possessed elevated levels of glial cell differentiation markers (GFAP) and neuron growth markers (NeuN, GAP-43, and NF200), respectively. C6 cells also showed higher levels of MAP2 and PSD-95 (differentiation and synaptic markers) in treated cultures. Taken together, these data endorsed their differentiation into astrocytic or neuron lineages, respectively, which was further confirmed by immunostaining of GFAP and NeuN (Figure 4D). The upregulation of mRNA expression of the above markers further supported the differentiation status in treated C6 and IMR-32 cells (Figure 4E).

**Figure 5:**
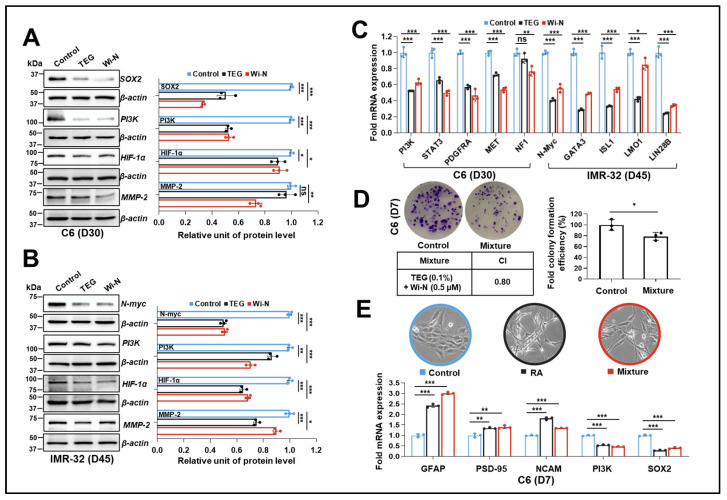
Reduction of cancer progression-related markers in differentiated C6 and IMR-32 cells induced by TEG (0.1%) and Wi-N (5 µM) treatment. (**A**,**B**) Western blotting analysis showing decreased levels of SOX2, PI3K, HIF-1α, and MMP-2 in differentiated C6 cells (**A**), as well as N-myc, PI3K, HIF-1α, and MMP-2 in differentiated IMR-32 cells (**B**). Quantification from three independent experiments is shown on the right side. The N-myc band was detected on the same blot of NeuN shown in Figure 4C. (**C**) RT-qPCR analysis showing downregulation of genes related to glioblastoma or neuroblastoma proliferation and metastasis in differentiated C6 and IMR-32 cells. (**D**) Colony formation assay shows a reduction in colony formation efficiency upon TEG (0.1%) + Wi-N (5 µM) mixture in C6 cells at day 7 exhibiting a synergistic effect as compared to each of the compounds alone (shown in Figure 2A). Quantification from three independent experiments is on the right side. (**E**) RT-qPCR analysis showing the effect of RA (7.5 µM) and TEG (0.1%) + Wi-N (5 µM) mixture on control and differentiated C6 cells at day 7. Morphological images under 10X magnification are displayed above the RT-qPCR results. For (**A**–**C**,**E**), data were normalized against the control group and plotted as fold difference (mean ± SD, n = 3). For (**D**), data were normalized against the control group and plotted as percentage difference (mean ± SD, n = 3). The combination index for the mixture was calculated for the individual compounds (shown in Figure 2A). ^ns^ *p* ≥ 0.05, * *p* < 0.05, ** *p* < 0.01, *** *p* < 0.001 denote statistical significant differences from the control group (**A**–**C**,**E**): one-way ANOVA with Dunnett’s multiple comparisons; (**D**): unpaired Student’s *t*-test). Control: 0.05% DMSO; ns: not significant; RA: retinoic acid; TEG: triethylene glycol; Wi-N: Withanone; Wi-A: Withaferin A.

We next subjected the differentiated cells to Western blotting to examine whether such differentiation induced by TEG or Wi-N triggered attenuation in their tumorigenic properties. As expected, a strong reduction in SOX2 and PI3K in differentiated C6 cells was observed (Figure 5A,C). Similarly, the differentiated IMR-32 cells showed downregulation of N-myc and PI3K (Figure 5B,C). Furthermore, expression of several genes associated with glioblastoma (*STAT3*, *PDGFRA*, *MET*, and *NF1*) and neuroblastoma (*GATA3*, *ISL1*, *LMO1*, and *LIN28B*) progression was downregulated at the transcriptional level in differentiated cells (Figure 5C). Of note, HIF-1α and MMP-2, both involved in malignant transformation and angiogenesis, showed a reduction in differentiated cells (Figure 5A,B). These data strongly supported the inhibition of stemness and tumorigenicity in differentiated cells. Next, following our previous study showing the enhanced antiaging efficacy of a mixture of TEG and Wi-N (0.1% and 5 µM, respectively) [36], we hypothesized that this mixture may show stronger pro-differentiation ability. Indeed, based on the results presented in Figure 5D, treatment of C6 cells with this mixture for 7 days resulted in approximately 20% inhibition of colony formation efficiency, compared to less than 10% inhibition observed with each compound individually (Figure 1). Combination index was calculated to be 0.80; suggesting that the TEG and Wi-N mixture exhibited a synergistic in vitro pharmacodynamic interaction. We next examined such an effect compared to the reported pro-differentiating agent RA [23]. As expected, the mixture-treated C6 cells showed strong differentiation after 7 days of culture, as evidenced by cell morphology, increased expression of *GFAP*, *PSD-95*, and *NCAM* mRNA, as well as decreased expression of *PI3K* and *SOX2* mRNA (Figure 5E). Of note, the effect was quantitatively equal to the effect of RA (Figure 5E). Overall, the above results demonstrated that TEG and Wi-N may inhibit cancer progression through the inhibition of cancer cell stemness and induction of differentiation.

## 3. Discussion

In recent decades, advances in understanding cancer and in technology have improved the outcomes of cancer therapy. However, the adverse effects of chemotherapy and the recurrence of tumors, especially in elderly patients, have highlighted the need for safer and more efficient therapeutic approaches that impose a lower treatment burden [2]. Stress can accelerate biological aging and increase the risk of cancer. Recent clinical trials have shown that stress management may reduce the recurrence of cancer and mortality [4]. In the present study, we included three antistress compounds (TEG, Wi-N, and Wi-A) identified in our previous research [36]. We evaluated their potential as agents for inhibiting cancer cell stemness and promoting differentiation therapy.

Metastasis is a major cause of treatment failure and cancer mortality. EMT, characterized by the loss of epithelial and upregulation of mesenchymal markers, promotes metastasis by activating signaling pathways such as Wnt and Notch [21]. We found that the cells treated with non-toxic doses of TEG, Wi-N, and Wi-A were compromised for their metastasis ability (Figure 1A,B and Figure S2A). Furthermore, the decrease in hnRNP-K and CARF, both of which can promote metastasis by activating EMT [41,42], further supported the inhibitory effect of TEG, Wi-N, and Wi-A on EMT (Figure 1C–F). Although the origin of CSCs is still debated, it has been closely linked to activated EMT signaling [14,22]. We found that in long-term cultures, TEG and Wi-N inhibited tumorigenic capacity by downregulating stemness-related genes both in MCF-7 and C6 cells (Figure 2B–D). Notably, such prolonged treatment of TEG and Wi-N did not trigger cell death, as reflected by the comparable colony number with the control group (Figure 2A). In contrast, Wi-A led to a decrease in clonogenicity (Figure 2A), which has also been observed in previous studies [37,43]. Analysis using Western blotting further indicated that extended treatment of MCF-7 and MDA-MB-231 cells with Wi-A resulted in a slight but significant decrease in the Bcl2/Bax ratio and c-Myc level compared to the control, which was not observed in Wi-N- or TEG-treated cells (Figure S3). These data suggested different modes of action between TEG, Wi-N, and Wi-A and relatively stronger cytotoxicity of the latter during long-term treatment [37]. Molecular understanding of such differential modes of action of these compounds warrants further in-depth analyses.

We also studied the impact of TEG and Wi-N on differentiation in three in vitro models, considering that promoting differentiation in cancer stem cells could be a potential treatment strategy for malignant cancers [23]. TEG and Wi-N induced a shift towards epithelial-like differentiation in experiments with breast carcinoma models (MCF-7 and MDA-MB-231 cells). This was evident from the cell cycle arrest supported by a decrease in Cyclin D1 and Cdk 4 and an increase in p27 and p21 (Figure 3A–C). The latter is engaged in multiple activities, including terminal differentiation and the inhibition of apoptosis [44]. We further validated the terminal differentiation by mRNA analysis of the molecular markers in control and treated cells. Figure 3D–E shows upregulation in epithelial/luminal markers (*KRT18*, *KRT19*, and *E-cadherin* mRNA) was observed in both MCF-7 and MDA-MB-231 cells. Of note, both MCF-7 and MDA-MB-231 cells did not show activated apoptosis upon TEG and Wi-N treatment, which could be attributed to upregulated p21 and p27, at least in part (Figure 3B,C). Additionally, TEG-treated MCF-7 cells also exhibited increased PPARγ at both protein (Figure 3F) and mRNA levels (Figure 3G), which was in line with the increase in p27, further supporting the inhibition of cell proliferation and induction of differentiation [45]. CD36, reported to regulate breast cancer progression and regarded as a potential therapeutic target [46], showed a reduction in treated MCF-7 cells (Figure 3H), further endorsing the differentiation of breast cancer cells. Notably, the metabolic changes in cancer cells, which are driven by c-Myc overexpression, are necessary to support the elevated need for nucleic acids, proteins, and lipids essential for rapid cell proliferation [47]. The above findings implied the potential effects of the three compounds on cancer metabolism reprogramming, which might be one of the mechanisms involved in the differentiation of cancer cells [48]. Furthermore, these compounds caused a reduction in stress-induced ROS production, offering another possible mechanism of their action [36].

The above findings suggested that low, non-toxic doses of TEG, Wi-N, and Wi-A inhibit EMT and stemness and induce differentiation in breast cancer cells by multiple mechanisms. In parallel, TEG and Wi-N induced astrocytic and neurodifferentiation in C6 glioblastoma and IMR-32 neuroblastoma, respectively (Figure 4). This was further strengthened by the reduction in markers involved in cancer progression (Figure 5A–C). Notably, such reduction was not observed at the initiative phase of differentiation (Figure S4), implying TEG and Wi-N may inhibit brain cancer progression by the induction of differentiation in a non-toxic manner.

It is important to note that a mixture of TEG and Wi-N demonstrated a synergistic effect, leading to significant differentiation that was quantitatively equivalent to RA (Figure 5D,E). These findings align with a previous report indicating that combining Ashwagandha water and alcoholic extracts (containing TEG and Wi-N as the primary bioactive components) exhibited a better pro-differentiation effect [49]. Notably, TEG and Wi-N show substantial structural differences. A previous study reported that TEG had been used to prepare gallic acid-trimethylene glycol dendrimer, which improved biomedical applications’ properties, such as drug and gene delivery [50]. We hypothesized that the mixture of TEG and Wi-N may form a combination with higher bioavailability, thereby exhibiting improved pro-differentiation efficacy. Our findings suggested a potential combinatorial approach to overcome the limitations of applying Wi-N related to its low bioavailability; however, further investigations are warranted.

Many independent studies have demonstrated the anticancer potentials of TEG, Wi-N, and Wi-A at their higher toxic doses. TEG was reported to cause selective toxicity to cancer cells by activating p53 and pRB signaling [38]. Wi-N triggered apoptosis by activating ROS signaling [51], disruption of TXP2-Aurora A complex [52], and mortalin-p53 complex [39] in cancer cells. In comparison, Wi-A has been reported to possess extensive anticancer activities mediated by modulation of p53 [40], Akt [53], Myc [54], NFκB [55,56], and Vimentin [57] signaling. Pharmacokinetic studies in mice revealed that Wi-A reaches peak concentrations of up to 2 µM in plasma following a single 4 mg/kg dose [57]. Other in vivo studies have reported that doses of 8 mg/kg [40] and 5 mg/kg [53] of Wi-A reduced nearly 70% and 25% of the tumor volume in nude mice, respectively. Our study found that despite causing less than 10% inhibition of cell viability, the three compounds significantly inhibited cancer stemness characteristics and induced terminal differentiation that offers advantages in non-toxic cancer treatment and prevention of its relapse.

Our present findings suggest a new aspect of the anticancer potential of TEG, Wi-N, and Wi-A. Specifically, we found that Wi-A primarily inhibits cancer cell proliferation, while TEG and Wi-N are more effective in controlling cancer metastasis and stemness. Consequently, it is suggested that combining these three compounds can be beneficial in cancer treatment and could also be considered as adjuvants with conventional chemotherapeutic drugs. Furthermore, it is anticipated that well-known antistress compounds such as natural antioxidants resveratrol and curcumin may work similarly and warrant further preclinic and clinical studies to promote their use in cancer treatment and prevention.

## 4. Materials and Methods

### 4.1. Cell Culture and Reagents

Human breast carcinoma MCF-7 (RRID: CVCL_0031) and MDA-MB-231(RRID: CVCL_0062), human neuroblastoma IMR-32 (RRID: CVCL_0346), and rat glioblastoma C6 (RRID: CVCL_0194) were obtained from the Japanese Collection of Research Bioresources (Tokyo, Japan). MCF-7, MDA-MB-231, and C6 were maintained in Dulbecco’s Modified Eagle Medium (DMEM; 041-29775, Fujifilm WAKO, Osaka, Japan) supplemented with 10% (*v*/*v*) fetal bovine serum (FBS; A5256701, Gibco BRL) and 1% (*v*/*v*) penicillin/streptomycin (168-23191, Fujifilm WAKO, Osaka, Japan) in a humidified incubator (37 °C and 5% CO_2_). IMR-32 cells were cultured in Eagle’s Minimum Essential Medium (EMEM; 051-07615, Fujifilm WAKO, Osaka, Japan) supplemented with 10% (*v*/*v*) FBS and 1% (*v*/*v*) penicillin/streptomycin on above standard conditions. Withanolides (Withanone, Wi-N, and Withaferin-A, Wi-A) present in Ashwagandha leaves were purchased from Tokiwa Phytochemical Co., Ltd. (Tokyo, Japan) and dissolved in dimethyl sulfoxide (DMSO; 043-07216, Fujifilm WAKO, Osaka, Japan) to prepare the 50 mM stock. Triethylene Glycol, TEG (T59455) was purchased from Sigma-Aldrich (Tokyo, Japan). The stock concentration of TEG was regarded as 100% (*v*/*v*), equivalent to 7.47M. The DMSO (0.05%) was used as a vehicle and marked as a control. Cells were authenticated using short tandem repeat (STR) profiling and screened for mycoplasma contamination by a mycoplasma detection kit (06495605001, Roche Applied Science, Mannheim, Germany).

### 4.2. Cell Viability Assay

Short-term cytotoxicity was detected by the 3-(4, 5-dimethylthiazol-2-yl)-2, 5-diphenyltetrazolium bromide (MTT) assay. Cells (5000 cells/well of 96-well plates) were treated with TEG, Wi-N, and Wi-A at varying concentrations at 24 h post-seeding. MTT (0.5 mg/mL, M2003, Sigma-Aldrich, St. Louis, MO, USA) was added at the assay’s endpoints (24 h, 48 h, and 72 h). The medium with MTT was replaced by 100 mL of DMSO after 4 h of incubation at 37 °C. The cell viability was determined as a percentage against the control group according to the absorbance measured at 570 nm using a microplate reader (Infinite M200 PRO, TECAN, Männedorf, Switzerland) [36].

### 4.3. Colony Formation Assay

The long-term cytotoxicity of TEG, Wi-N, and Wi-A was determined by colony formation assay. Cells (500 cells/well of 6-well plates) were seeded and allowed to adhere overnight, followed by treatment with 0.1% (7.47 mM) TEG, 5 µM Wi-N, 0.1 µM Wi-A, or the mixture of TEG (0.1%) and Wi-N (5 µM) for 7 days. The control and test compound-supplemented media were changed every alternate day. Colonies were washed with cold phosphate-buffered saline (PBS) and fixed with pre-chilled acetone: methanol (1/1, *v*/*v*) at 4 °C for 10 min, washed with cold PBS, and stained with 0.1% crystal violet solution. The dishes were washed with water and left open for drying, followed by photography and colony counting. The colony number and size of each group were analyzed using ImageJ software (version 1.54, NIH, Bethesda, MD, USA) [36].

### 4.4. Wound-Healing Assay

Cells (2 × 10^5^ cells/well of 6-well plates) were seeded and allowed to adhere and form a monolayer, following which a wound was created by scratching the monolayer with a 200 µL-pipette. Cells were washed with PBS and continued to culture in a control or test compound-supplemented serum-free medium. The movement of cells to the scratched area was periodically monitored and captured using a phase-contrast microscope (Nikon Eclipse TE300, Nikon, Tokyo, Japan) at 4× magnification. The migration ability of the cells was reflected as the relative unit of wound area to time point 0 h in three independent experiments [43].

### 4.5. Transwell Invasion Assay

The invasion ability of control and treated cells was evaluated using a cell invasion assay kit (ECM550, Sigma-Aldrich) following the manufacturer’s instructions. Briefly, bovine serum was added to the wells of the 24-well culture plate as the chemoattractant, followed by cell suspension in the chambers. Dishes were incubated in a humidified incubator (37 °C and 5% CO_2_) for 48 h. The invasive cells on the lower surface of the insert membrane were fixed and stained by staining solution. Images of invasive cells were taken under a microscope at 10X magnification. Invaded cells were quantitated by dissolving the stained cells in 10% acetic acid for colorimetric reading of OD at 560 nm.

### 4.6. Tumorsphere Formation Assay

Cells were suspended in Cancer Stem Cell Medium (C-28070, PromoCell, Heidelberg, Germany) supplemented with TEG (0.1%), Wi-N (5 µM) or Wi-A (0.1 µM) and seeded at 1 × 10^5^ cells/well in Ultra-low Attachment 6-well Plates (3471, Corning, New York, NY, USA). The cells were incubated at 37 °C in a humidified incubator for 7 days (MCF-7) or 10 days (C6) and regularly watched for tumorsphere formation and progress. The tumorspheres with diameters ≥ 150 μm (MCF-7) or ≥100 μm (C6) were considered positive, and the number of positive spheres was counted under a microscope. At least 50 spheres were measured for each group, and the tumorsphere formation efficiency was calculated as the number of positive spheres divided by the total number of spheres from three independent experiments [54].

### 4.7. Extreme Limiting Dilution Assay (ELDA)

Tumorspheres treated with the three test compounds were collected and trypsinized. The cells were centrifuged and re-suspended in Cancer Stem Cell Medium (C-28070, PromoCell) and passed through a 40 μm cell strainer (352340, Corning) to make a single-cell suspension. Cells were then seeded at gradient densities (1, 50, 100, and 500 cells/well) in Ultra-low Attachment 96-well Plates (7007, Corning). Each condition was replicated in 24 wells. After 7 days of incubation, the amount of spheroid with a size bigger than 150 μm (MCF-7) or 50 μm (C6) was counted, and the frequency of spheroid initiation capacity was calculated by ELDA [58].

### 4.8. Cell Differentiation and Morphology Observation

Cells (5 × 10^4^ cells/mL) were seeded in 6 cm dishes and treated with the indicated compounds (0.1% TEG, 5 µM Wi-N, 0.1 µM Wi-A, 7.5 µM retinoic acid (RA, a positive control for differentiation), or the mixture of 0.1% TEG and 5 µM Wi-N). The control and compound-supplemented medium were replaced every alternate day for up to 45 days. During the long-term cultures, neither the control nor the treated cells showed abrupt cell death. The cells were passaged at a ratio of 1:3 (C6, MCF-7, and MDA-MB-231) or 1:2 (IMR-32) till the differentiated morphology was observed, followed by harvesting or re-seeding for molecular assays. The morphology of control and treated cells was recorded with a phase-contrast microscope (Nikon Eclipse TE300, Nikon, Tokyo, Japan) at 10× magnification.

### 4.9. Combination Index (CI) Analysis

The CI value was calculated using CompuSyn software based on the Chou-Talalay method [59]. The drug interactions were determined as synergism (CI < 1), additive effect (CI = 1), and antagonism (CI > 1).

### 4.10. Immunoblotting

The control and treated cells were lysed in radio-immunoprecipitation assay buffer (188-02453, Fujifilm WAKO, Osaka, Japan) containing protease inhibitor (11697498001, Roche Applied Science) at 4 °C for 30 min to extract protein. The protein amount was determined using the Pierce BCA Protein Assay Kit (23250, Thermo Fisher Scientific, Waltham, MA, USA). Lysate containing 20 mg of protein was separated using SDS–polyacrylamide gel electrophoresis and then transferred to a polyvinylidene difluoride membrane (IPVH00010, Millipore, Billerica, MA, USA). The membranes were blocked by shaking in 3% bovine serum albumin (BSA; 011-15144, Fujifilm WAKO) at room temperature for 1 h and then incubated with specific primary antibodies (listed in Table S1) at 4 °C overnight, followed by incubation with secondary antibodies including anti-rabbit IgG (31460, Thermo Fisher Scientific) or anti-mouse IgG (31340, Thermo Fisher Scientific) at room temperature for 1 h. Internal controls were β-actin (643807, Biolegend, San Diego, CA, USA) and α-Tubulin (T5168, Sigma-Aldrich, St. Louis, MO, USA). Protein bands were detected by Gel Doc Documentation (Bio-Rad, Hercules, CA, USA), and the protein level from three independent experiments was analyzed using ImageJ software (NIH, Bethesda, MD, USA).

### 4.11. Immunostaining

The control and treated cells were seeded on 18 mm glass coverslips placed in 12-well plates and allowed to adhere. The cells were washed with cold PBS and then fixed with 4% paraformaldehyde at room temperature for 30 min. Fixed cells were washed with PBS and then permeabilized by incubating with PBS-Triton X-100 (0.1%) for 10 min, followed by blocking with 2% BSA at room temperature for 1 h. The coverslips were incubated with a specific primary antibody (as indicated in the results and listed in Table S1) at 4 °C overnight. Secondary antibodies conjugated with Alexa-488 or Alexa-594 (Molecular Probes, Eugene, OR, USA) were added for 1 h. Hoechst 33342 (1 μg/mL, H3570, Thermo Fisher Scientific) was used for nuclear staining. The cells stained with secondary antibodies alone were recruited as negative control. The coverslips were mounted and visualized under a Carl Zeiss microscope (Axiovert 200M, Tokyo, Japan) with an exposure time of 500 ms. The protein expression level was quantified using ImageJ software (NIH, Bethesda, MD, USA).

### 4.12. RNA Extraction and Reverse Transcription–Quantitative Polymerase Chain Reaction (RT-qPCR)

The control and treated cells were harvested, and the total RNA was extracted using an RNeasy mini kit (74104, Qiagen, Venlo, The Netherlands) following the manufacturer’s protocol. RNA (1 μg) from each group was reverse transcribed into cDNA using the QuantiTect Rev Transcription Kit (205311, Qiagen). cDNA was then subjected to RT-qPCR using SYBR Select Master Mix (4472908, Applied Biosystems, Foster City, CA, USA) with primers (listed in Table S2) on the Eco real-time system (Illumina, San Diego, CA, USA). The PCR amplification conditions consisted of 50 °C for 2 min, 95 °C for 10 min, followed by 40 cycles at 95 °C for 15 sec and 60 °C for 1 min. The housekeeping gene 18S was used as an internal control to normalize the variability in expression levels.

### 4.13. Statistical Analysis

The obtained data were normalized against the control group and expressed as the mean ± standard deviation (SD) value of three experimental sets. Statistical significance was evaluated by one-way ANOVA with Dunnett’s multiple comparisons or unpaired Student’s *t*-test using GraphPad software and shown as ^ns^ *p* ≥ 0.05, * *p* < 0.05, ** *p* < 0.01, and *** *p* < 0.001.

## 5. Conclusions

**Figure 6:**
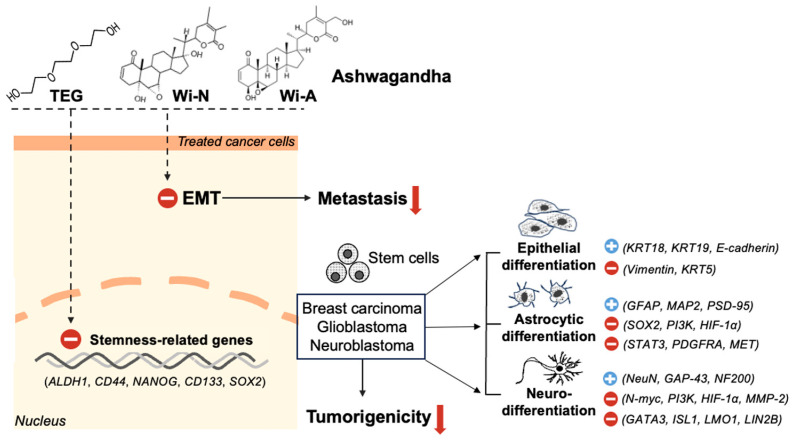
Graphical representation of the main findings of the current study. It depicts the structures of the three compounds and their molecular effects on protein markers associated with stemness and differentiation (epithelial, astrocytic, and neuronal). The symbol (+) indicates an increase, while (−) signifies a decrease in cells treated with TEG and Wi-N.

Antistress compounds TEG, Wi-N, and Wi-A caused a reduction in metastatic and self-renewal properties of cancer cells by inhibiting EMT and stemness signaling. TEG and Wi-N caused strong inhibition of stemness and induction of differentiation in breast carcinoma, glioblastoma, and neuroblastoma cells in a non-toxic manner and hence are predicted to benefit cancer treatment (Figure 6). Further studies are warranted to promote their clinical use and adjuvants to conventional therapies.

## Data Availability

The manuscript and Supplementary Information files contain all datasets used and/or analyzed during the current study.

## Supplementary Materials

The following supporting information can be downloaded at: https://www.mdpi.com/article/10.3390/ijms26020493/s1. Reference [60] is cited in the supplementary materials.

## Abbreviations

ADBR2: β2-adrenergic receptor; BDNF: brain-derived neurotrophic factor; BRD4: bromodomain-containing protein 4; BSA: bovine serum albumin; CARF: Collaborator of ARF; CI: combination index; CSCs: cancer stem cells; DMEM: Dulbecco’s Modified Eagle Medium; DMSO: dimethyl sulfoxide; ELDA: extreme limiting dilution assay; EMEM: Eagle’s Minimum Essential Medium; EMT: Epithelial–mesenchymal transition; FBS: fetal bovine serum; HDAC: histone deacetylases; hnRNP-K: heterogeneous nuclear ribonucleoprotein K; IDH: isocitrate dehydrogenase; MMP: matrix metalloproteinase; MRPs: multidrug resistance proteins; MTT: 3-(4, 5-dimethylthiazol-2-yl)-2, 5-diphenyltetrazolium bromide; NGF: nerve growth factor; PBS: phosphate-buffered saline; PPARγ: peroxisomal proliferator receptor-γ; RA: retinoic acid; ROS: reactive oxygen species; RT-qPCR: reverse transcription–quantitative polymerase chain reaction; SD: standard deviation; TEG: triethylene glycol; Wi-A: Withaferin A; Wi-N: Withanone.
