# Supplementary material for: Molecular Characterization of Cancer Preventive and Therapeutic Potential of Three Antistress Compounds, Triethylene Glycol, Withanone, and Withaferin A
Source: Int J Mol Sci. 2025 Jan 9;26(2):493. doi: 10.3390/ijms26020493 (PMC11764651; doi:10.3390/ijms26020493)
Supplement: Supplementary file 1 [file ijms-26-00493-s001.zip › ijms-3389002-Supplementary.pdf]

SUPPLEMENTARY INFORMATION

Molecular characterization of cancer preventive and therapeutic potential of three antistress compounds, triethylene glycol, Withanone, and Withaferin A

Huayue Zhang[1,2], Hyonchol Kim[2], Tian Yuan[1], Zhenya Zhang[1], Sunil C Kaul[2], and Renu Wadhwa[2]*

[1]Graduate School of Science and Technology, University of Tsukuba, Ibaraki 305-8575, Japan

[2]AIST-INDIA DAILAB, National Institute of Advanced Industrial, Science & Technology (AIST), Central 4-1, Tsukuba 305-8565, Japan

*Corresponding author: Renu Wadhwa

E-mail: renu-wadhwa@aist.go.jp

Supplementary Table S1-S2 and Figure S1-S4

Table S1. List of primary antibodies used in Western blotting.

**Antibody**	**Species**	**Source**	**Cat. No.**	**Antibody**	**Species**	**Source**	**Cat. No.**
Wnt-1	Mouse	Santa Cruz	sc-514531	Bcl-2	Rabbit	Abcam	ab196495
MMP-3/10	Mouse	Santa Cruz	sc-374029	Bax	Mouse	Santa Cruz	sc-7480
MMP-2	Mouse	Santa Cruz	sc-13594	caspase-3	Mouse	Santa Cruz	sc-7272
β-catenin	Mouse	Santa Cruz	sc-7963	c-Myc	Rabbit	Abcam	ab32072
hnRNP-K	Rabbit	Cell Signaling	4675	p21 (For C6 cells)	Rabbit	Santa Cruz	sc-397
E-cadherin	Mouse	Cell Signaling	14472	Sox-2	Mouse	Santa Cruz	sc-365823
N-cadherin	Rabbit	Cell Signaling	13116	GFAP	Rabbit	Sigma	G9269
Vimentin	Mouse	Santa Cruz	sc-6260	PI3K	Rabbit	Cell Signaling	4249
CARF	Rabbit	Raised in our laboratory [60]	----	N-Myc	Mouse	Santa Cruz	sc-53993
Cyclin D1	Mouse	Santa Cruz	sc-56302	GAP-43	Mouse	Santa Cruz	sc-33705
p27	Mouse	Santa Cruz	sc-1641	NF200	Mouse	Cell Signaling	2836
Cdk4	Rabbit	Santa Cruz	sc-260	MAP2	Rabbit	Cell Signaling	4542
p21 (For human cell lines)	Rabbit	Cell Signaling	2947	PSD-95	Mouse	Santa Cruz	sc-32290
PPARγ	Rabbit	Cell Signaling	2435	NeuN	Rabbit	Abcam	ab177487
p53	Mouse	Santa Cruz	sc-126	HIF-1α	Rabbit	Novus Biologicals	NB100-479

Table S2. Sequence of primers used in RT-qPCR.

Cell line	Gene	Forward primer	Reverse primer
MCF-7	*ALDH1*	TCGTCTGCTGCTGGCGACAATG	CCCAACCTGCACAGTAGCGCAA
	CD44	TGGCACCCGCTATGTCGAG	GTAGCAGGGATTCTGTCTG
	NANOG	TTTGTGGGCCTGAAGAAAACT	AGGGCTGTCCTGAATAAGCAG
MDA-MB-231	*KRT5*	TGTTCTTTGATGCGGAGCTG	TTGTCCATGGAGAGGACCAC
MCF-7 & MDA-MB-231	*KRT18*	GCTGGAAGATGGCGAGGACTTT	TGGTCTCAGACACCACTTTGCC
	KRT19	AGCTAGAGGTGAAGATCCGCGA	GCAGGACAATCCTGGAGTTCTC
	E-cadherin	CGGGAATGCAGTTGAGGATC	AGGATGGTGTAAGCGATGGC
	vimentin	CCTTGAACGCAAAGTGGAATC	GACATGCTGTTCCTGAATCTGAG
	PPARγ	TTGAAAGAAGCCAACACTAAACCAC	AATGGCATCTCTGTGTCAACCAT
	CD36	TGGAACAGAGGCTGACAACTT	TTGATTTTGATAGATATGGGATGC
C6	*SOX2*	TACAGCATGTCCTACTCGCAG	GAGGAAGAGGTAACCACAGGG
	CD44	AAGACATCGATGCCTCAAAC	CTCCAGTAGGCTGTGAAGTG
	CD133	CCAGCGGCAGAAGCAGAACGA	GTCAGGAGAGCCCGCAAGTCT
	GFAP	TTGCAGACCTCACAGACGTT	AGTTGGCGGCGATAGTCATT
	PSD-95	TCTGTGCGAGAGGTAGCAGA	AAGCACTCCGTGAACTCCTG

Table S2. Continued Sequence of primers used for RT-qPCR.

C6	*MAP2*	ACATCCTCCGAGTCACCCTT	CCTCAGCATCTGCACTCACA
	PI3K	CACCTGAACAGACAAGTAGAGGC	GCAAAGCATCCATGAAGTCTGGC
	STAT3	GCTGGAACAGCATCTTCAGG	CTGTCTGGTCACAGACTGGT
	PDGFRA	GAGACGGGTTCCAGTAGTTCCACTT	CACCAGGTCTGAGGAATCTATGCCA
	MET	CCCTCCTTATCCTGACGTGAACACA	ACTTCATACAAGGCGTCTGGACAGT
	NF-1	CATTCCCCAGGAATCGACAAGGAGA	TTACGTTTGAAACTGCCAGCACTCC
	NCAM	GAATGTACCACCCACTGTCC	TGGGTTCCCCATCCTTTGTC
IMR-32	*GAP43*	TTCTTGGTGTTGTTATGGCAAG	GAGGAAAGTGGACTCCCACAG
	NF200	CAAGGAACCCAGCAAACCA	GGCCTCTGTCTTGGGTTTCTC
	NeuN	TACGCAGCCTACAGATACGCTC	TGGTTCCAATGCTGTAGGTCGC
	N-Myc	TCCATGACAGCGCTAAACGTT	GGAACACACAAGGTGACTTCAACA
	GATA3	ACCACAACCACACTCTGGAGGA	TCGGTTTCTGGTCTGGATGCCT
	ISL1	AAGGACAAGAAGCGAAGCAT	TTCCTGTCATCCCCTGGATA
	LMO1	TCTGCTGAAGGCATTGGA	TCGGCACAGGATGAGGTT
	LIN28B	ATATCGGTGTGCTGTGATGC	TGAGCAACGCTTATCATGTTTT
All	*18S*	CAGGGTTCGATTCCGTAGAG	CCTCCAGTGGATCCTCGTTA

Figure S1 MTT assay showing time (up to 72 h)- and dose-dependent cytotoxicity of TEG, Wi-N, and Wi-A in MCF-7 (A) and C6 (B) cells. For further experiments, 0.1% of TEG, 5 μM of Wi-N, and 0.1 μM of Wi-A were selected as non-toxic doses. All data was normalized against the control group (0.05% DMSO) and plotted as percentage difference (mean ± SD, n = 3). $^{ns}p \geq 0.05$, $^{*}p < 0.05$, $^{**}p < 0.01$, $^{***}p < 0.001$ denote statistical significance different from the control group (one-way ANOVA with Dunnett's multiple comparisons). ns: not significant; TEG: triethylene glycol; Wi-N: Withanone; Wi-A: Withaferin A.

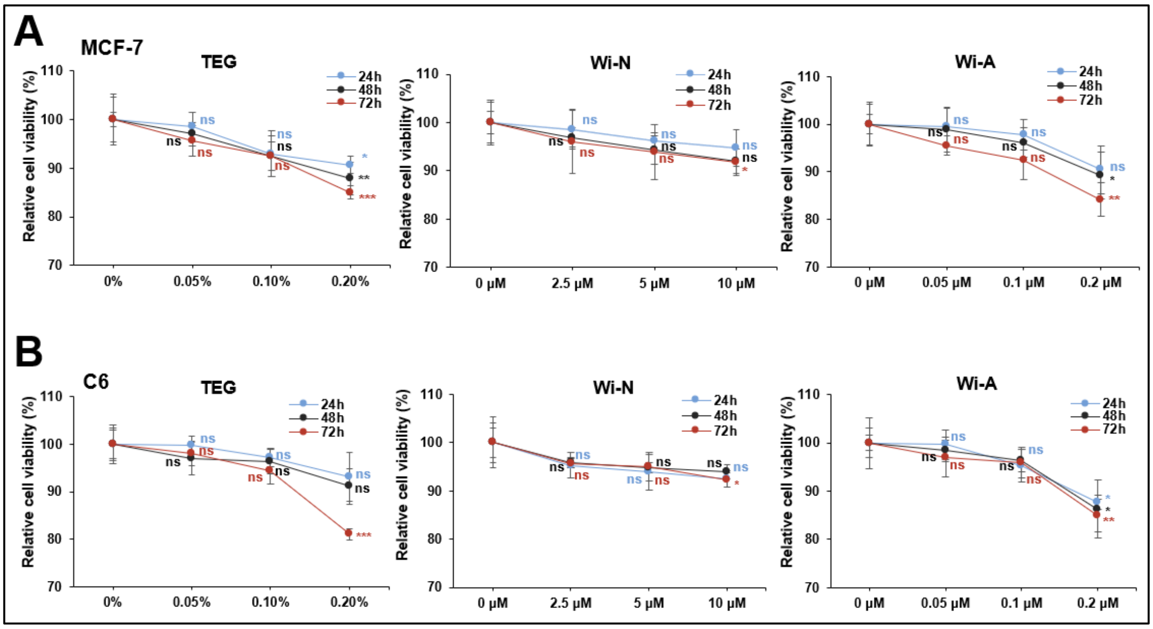

Figure S2 Effect of TEG (0.1%), Wi-N (5 μM) and Wi-A (0.1 μM) on migration and EMT signaling in MDA-MB-231 cells. (A) Wound healing assay shows decreased migration ability in TEG-treated MDA-MB-231 cells at 48 h. Images were captured at 4X magnification. Quantitation from three independent experiments is shown on the right side. (B) Western blotting analysis shows reduced vimentin level by TEG, Wi-N, and Wi-A treatment in MDA-MB-231 cells at 48 h. For (A), data was normalized against the wound area of 0 h of each group. For (B), data was normalized against the control group. All the data was plotted as fold difference (mean ± SD, n = 3). $^{ns}p \geq 0.05$ and $^{*}p < 0.05$ denote statistical significance different from the control group (one-way ANOVA with Dunnett's multiple comparisons). Control: 0.05% DMSO. ns: not significant; TEG: triethylene glycol; Wi-N: Withanone; Wi-A: Withaferin A.

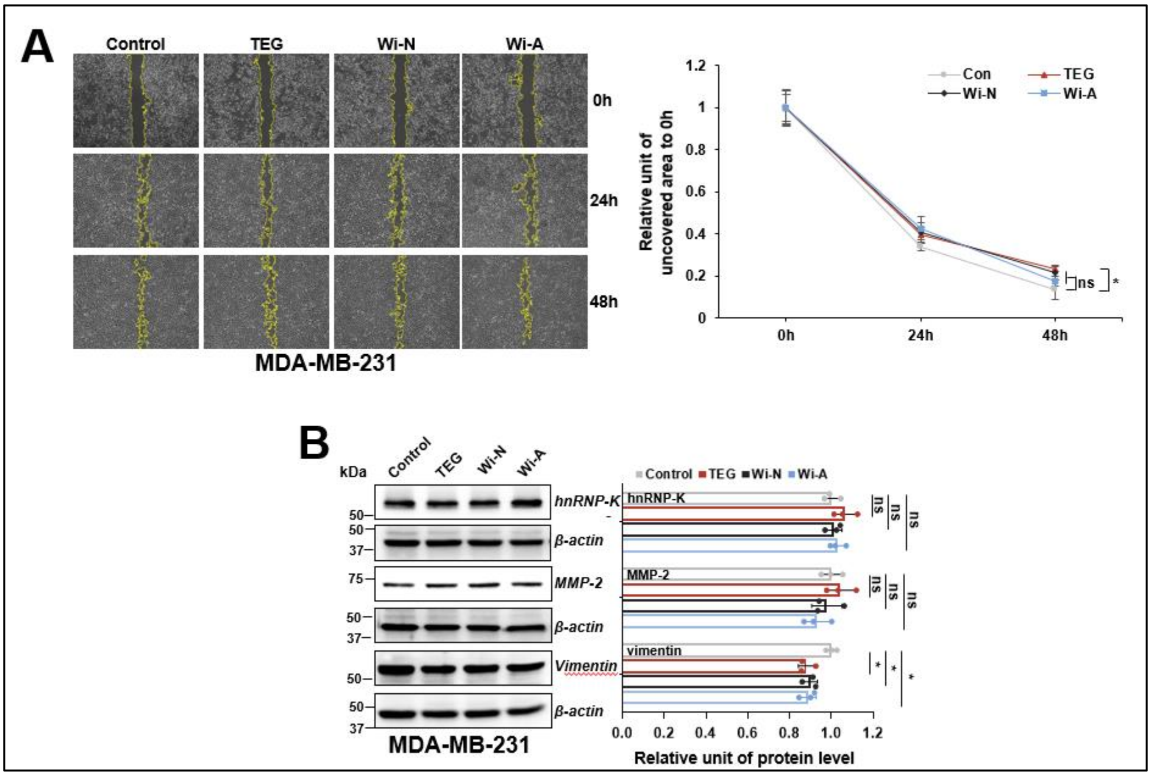

Figure S3 Western blotting shows initiation of mild apoptosis signaling by Wi-A (0.1 μM) in MCF7 and MDA-MB-231 cells after 30 days of culture but not by TEG (0.1%) or Wi-N (5 μM). Quantification from three independent experiments is shown on the right side. Data was normalized against the control group and plotted as fold difference (mean ± SD, n = 3). $^{ns}p \geq 0.05$, $^{*}p < 0.05$, $^{**}p < 0.01$, $^{***}p < 0.001$ denote statistical significance different from the control group (one-way ANOVA with Dunnett's multiple comparisons). Control: 0.05% DMSO. ns: not significant; TEG: triethylene glycol; Wi-N: Withanone; Wi-A: Withaferin A.

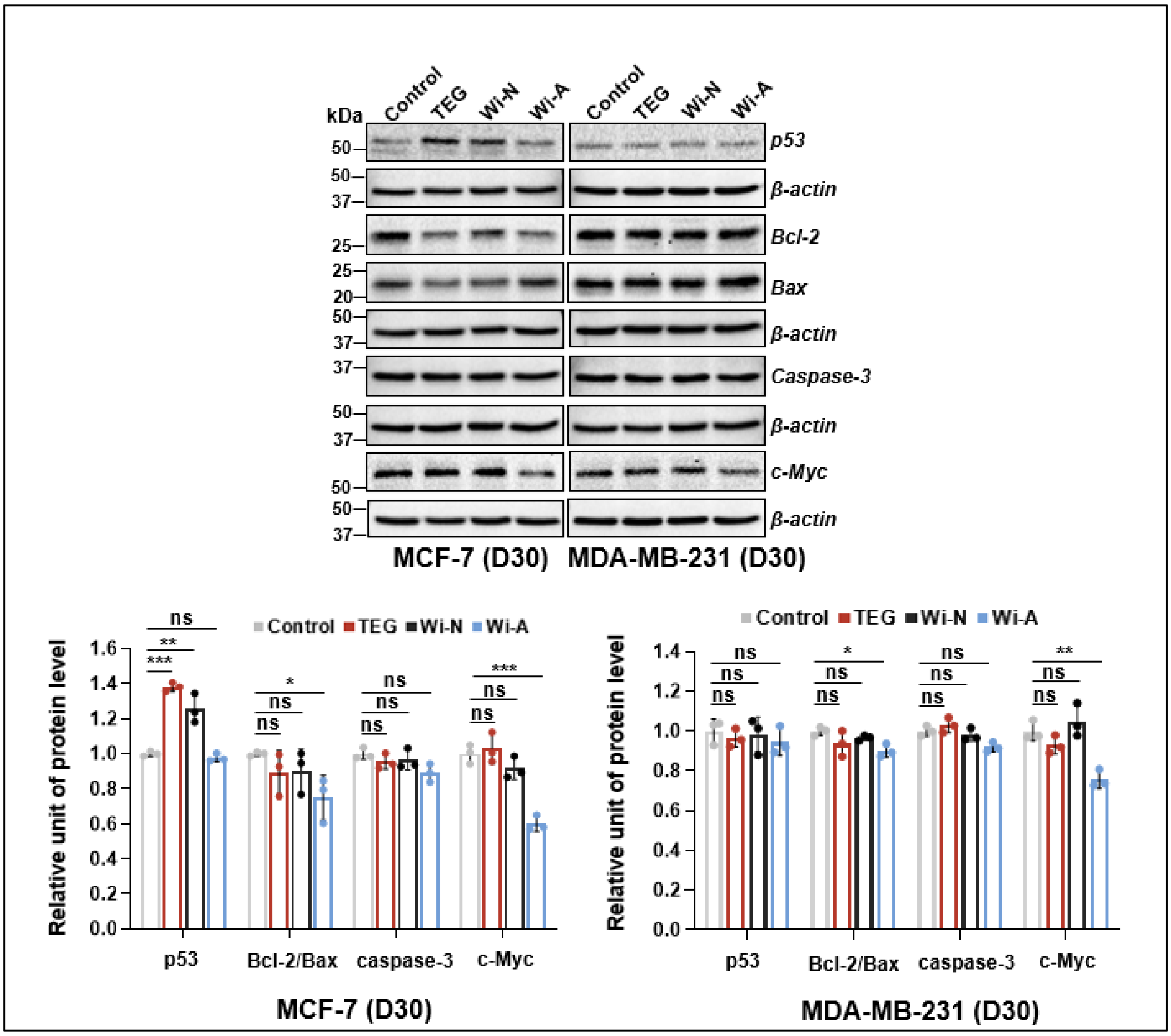

Figure S4 C6 cells showing faster response to TEG (0.1%) and Wi-N (5 µM) treatment than IMR-32 cells in terms of short-term cultures (96 h). (A and B), Western blotting analysis showed increased p21 and GFAP protein levels in treated C6 cells (A) but not in IMR-32 cells (B). Quantification from three independent experiments is shown on the right side. Data was normalized against the control group and plotted as fold difference (mean ± SD, n = 3). $^{ns}p \geq 0.05$, $^{**}p < 0.01$, $^{***}p < 0.001$ denote statistical significance different from the control group (one-way ANOVA with Dunnett's multiple comparisons). Control: 0.05% DMSO. ns: not significant; TEG: triethylene glycol; Wi-N: Withanone; Wi-A: Withaferin A

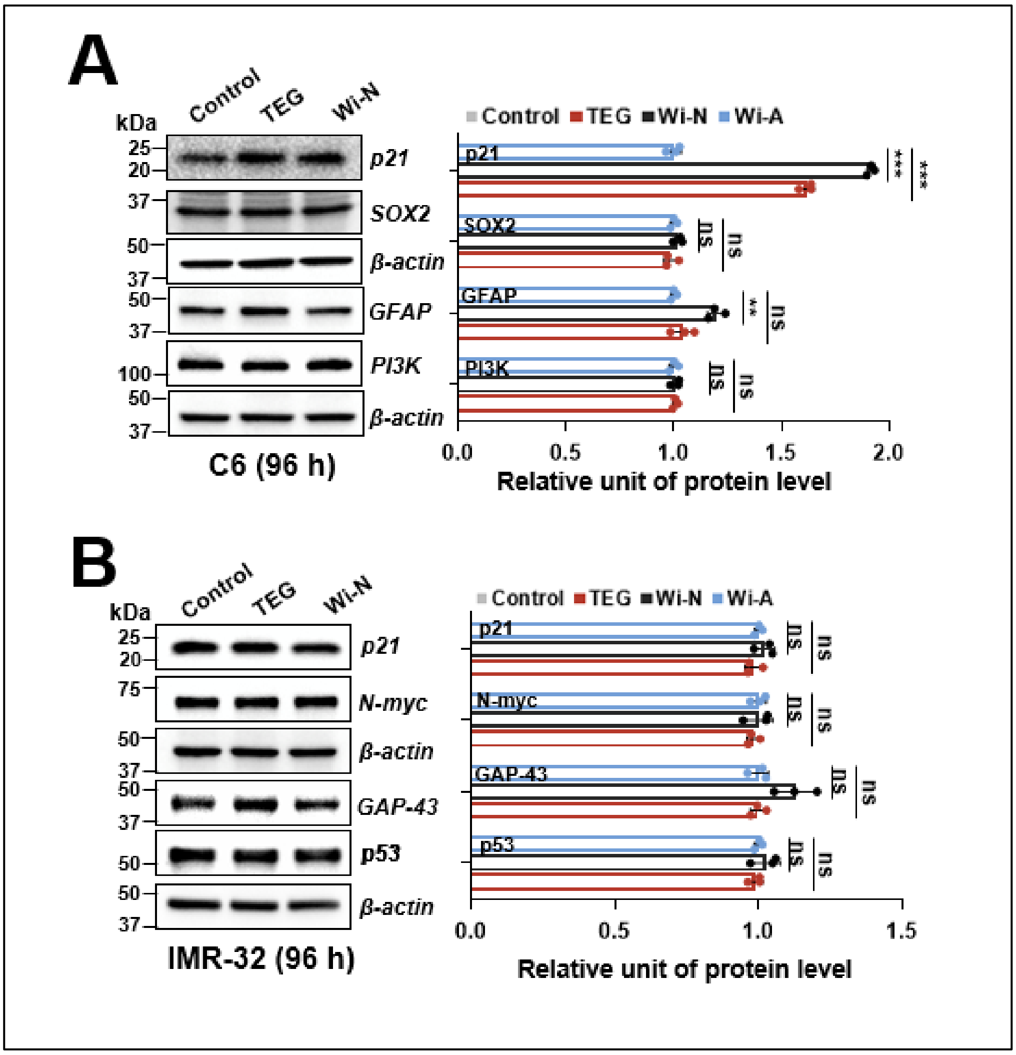

Supplementary reference

[60] Hasan, M.K.; Yaguchi, T.; Sugihara, T.; Kumar, P.K.; Taira, K.; Reddel, R.R.; Kaul, S.C.; Wadhwa, R. CARF is a novel protein that cooperates with mouse p19ARF (human p14ARF) in activating p53. J. Biol. Chem. 2002, 277, 37765 – 37770. https://doi.org/10.1074/jbc.M204177200.
